# *Giardia duodenalis*-induced G0/G1 intestinal epithelial cell cycle arrest and apoptosis involve activation of endoplasmic reticulum stress *in vitro*

**DOI:** 10.3389/fimmu.2023.1127552

**Published:** 2023-03-15

**Authors:** Shuyuan Yu, Huimin Zhao, Xuening Qin, Xiaoyun Li, Jiaying Guo, Wei Li

**Affiliations:** Heilongjiang Provincial Key Laboratory of Zoonosis, College of Veterinary Medicine, Northeast Agricultural University, Harbin, China

**Keywords:** *Giardia duodenalis*, IECs, UPR, ER stress, cell cycle arrest, apoptosis

## Abstract

*Giardia duodenalis* is a zoonotic intestinal protozoan parasite that may cause host diarrhea and chronic gastroenteritis, resulting in great economic losses annually and representing a significant public health burden across the world. However, thus far, our knowledge on the pathogenesis of *Giardia* and the related host cell responses is still extensively limited. The aim of this study is to assess the role of endoplasmic reticulum (ER) stress in regulating G0/G1 cell cycle arrest and apoptosis during *in vitro* infection of intestinal epithelial cells (IECs) with *Giardia*. The results showed that the mRNA levels of ER chaperone proteins and ER-associated degradation genes were increased and the expression levels of the main unfolded protein response (UPR)-related proteins (GRP78, p-PERK, ATF4, CHOP, p-IRE1, XBP1s and ATF6) were increased upon *Giardia* exposure. In addition, cell cycle arrest was determined to be induced by UPR signaling pathways (IRE1, PERK and ATF6) through upregulation of p21 and p27 levels and promotion of E2F1-RB complex formation. Upregulation of p21 and p27 expression was shown to be related to Ufd1-Skp2 signaling. Therefore, the cell cycle arrest was induced by ER stress when infected with *Giardia*. Furthermore, the apoptosis of the host cell was also assessed after exposure to *Giardia*. The results indicated that apoptosis would be promoted by UPR signaling (PERK and ATF6), but would be suppressed by the hyperphosphorylation of AKT and hypophosphorylation of JNK that were modulated by IRE1 pathway. Taken together, both of the cell cycle arrest and apoptosis of IECs induced by *Giardia* exposure involved the activation of the UPR signaling. The findings of this study will deepen our understanding of the pathogenesis of *Giardia* and the associated regulatory network.

## Introduction

The endoplasmic reticulum (ER) is the largest organelle in host cells and plays an important role in protein synthesis, transport, and folding. In response to ER stress, deregulation in ER homeostasis can trigger unfolded protein response (UPR) pathways with the aim of restoring homeostasis by activating genes involved in protein folding and antioxidants (1). In mammals, the UPR comprises three signaling pathways that regulated downstream of the ER membrane proteins IRE1, ATF6, and PERK (2). Upon activation, IRE1 splices x‐box binding protein 1 (XBP1) mRNA, which is then translated into XBP1s. Activated ATF6 is translocated to the Golgi. PERK‐mediated phosphorylation of eIF2α prompts selective translation of ATF4 (3). UPR upregulates the folding machinery by inducing ER chaperone genes to buffer ER stress and restore ER function (4). The ER-associated degradation (ERAD) process removes unfolded proteins to the cytosol where they are ubiquitinated and degraded (5). A moderate and mild ER stress induces UPR signaling as a compensatory mechanism, while a severe and chronically prolonged ER stress deteriorates the cellular functions and switches from an adaptation mode to death mode (4). The activated UPR involves various signaling pathways that in great part determine the fate and state of the cell (such as cell cycle, apoptosis, autophagy, inflammation, and senescence) (1). It has been previously shown that UPR activation regulates cell cycle progression and apoptosis (6–10).

Progression through the mammalian cell cycle is driven by the sequential activation of cyclin/cyclin dependent kinase (CDK) complexes (11). CDK activity requires binding of regulatory subunits known as cyclins. Cyclins are synthesized and destroyed at specific times during the cell cycle, thus regulating kinase activity in a timely manner (12, 13). Driving the cell cycle cyclin-CDK complexes include four CDKs (CDK1, CDK2, CDK4 and CDK6) and four different classes cyclins (cyclin A, cyclin B, cyclin D and cyclin E) (12). The Cip/Kip proteins (p21, p27 and p57) inhibit CDK activities at the G1/S and G2/M checkpoints (14, 15). E2F transcription factors are known to be important for timely activation of G1/S and G2/M genes required for cell cycle progression (16). Members of the E2F family are generally associated with transcriptional activation (E2F1, E2F2 and E2F3α) or repression (E2F4, E2F5, E2F6, E2F7 and E2F8) (17). Retinoblastoma protein (RB) is a tumor suppressor that represses the expression of E2F regulated genes required for cell cycle progression. A significant aspect of RB-E2F complexes is the switch from activating to repressing E2F promoter sites (18). In many cases, RB-E2F target genes affect the cell cycle by controlling DNA replication and G1-S transitions (18). Multiple parasite species, such as *Toxoplasma gondii*, *Eimeria tenella* and *Leishmania* spp. inhibit host cell proliferation by causing cell cycle arrest (19–21).

Apoptosis, also called programmed cell death, is not only a physical process that takes place in the development and maintenance of homeostasis in an organism, but also plays a crucial role in several pathological processes. Apoptosis can be triggered by either intrinsic or extrinsic pathways, depending on the type of cell and external factors (22, 23). Caspase (CASP) proteins function vitally in apoptosis. Activation of CASP3 is necessary for the activation of all apoptotic signaling pathways in the process of apoptosis. Poly (ADP-ribose) polymerase (PARP), a nuclear enzyme involved in DNA repair, a target of caspases during apoptosis. Inactivated PARP is regarded as an apoptosis marker (22). Some protozoan parasites such as *Blastocystis*, *Entamoeba histolytica* and *Leishmania donovani* utilize apoptosis mechanisms to facilitate infections within the host cell (24–26).

*Giardia duodenalis*, a microaerophilic zoonotic protozoan parasite, causes intestinal infections in humans and nonhuman animals (27). It is ingested by animals with the form of cysts and transformed to trophozoites when passing through the stomach and small intestine. After exposure to biliary fluid, some of trophozoites form cysts and are shed by infected hosts into the environment (28). The clinical presentations of human giardiasis typically include diarrhea, abdominal cramps, gas, weight loss or malabsorption, and asymptomatic infections are also common (29). Noninvasive *Giardia* infection is able to increase epithelial permeability and facilitates the invasion of gut bacteria, which causes chronic post-infectious gastrointestinal complications (30). Previous studies have documented that *Giardia* infection can induce the host cell cycle arrest and apoptosis (31–34), but the detailed mechanisms remain to be investigated and elucidated. More researches are needed to elucidate the complex interactions between host UPR signaling and infections by protozoan parasites (35).

To the best of our knowledge, current studies are insufficient to precisely explain the mechanisms of cell cycle arrest and apoptosis induced by *Giardia*. The aim of the present study is to explore the involvement of UPR signaling in *Giardia*-induced intestinal epithelial cell cycle arrest and apoptosis.

## Materials and methods

### Cell culture

The Caco-2 and HT-29 cell lines were gained from Cell Bank of the Chinese Academy of Sciences (Shanghai, China) and maintained in a humidified atmosphere at 37°C with 5% CO_2_ in this study. Caco-2 cells were grown in DMEM (Hyclone, Logan, UT, USA) supplemented with 10% fetal bovine serum (FBS; Cellmax, Beijing, China), 1% nonessential amino acids (Alphabio, Tianjin, China), 1% glutamine (Alphabio, Tianjin, China), and 1% penicillin/streptomycin. HT-29 cells were cultured in DMEM/F12 (Hyclone, Logan, UT, USA) supplemented with 10% FBS and 1% penicillin/streptomycin.

### Parasite culture

The *Giardia duodenalis* WB isolate typed as assemblage A was used in this study (ATCC30957, Manassas, VA, USA). *Giardia* trophozoites were grown in sterilized modified TYI-S-33 medium with 10% FBS, 0.1% bovine bile, 0.1% gentamycin and 1% penicillin/streptomycin at 37°C under microaerophilic conditions. *Giardia* was collected by replacing the culturing medium with fresh medium to eliminate dead parasites. The tubes were placed on ice for 10 to 20 minutes, centrifuged (2000 × g for 8 min at 4°C) for parasite collection. Trophozoites were then counted and diluted.

### Cell viability assay

CCK-8 (K1018; ApexBio, Houston, TX, USA) assay was performed according to the manufacturer’s instructions to assess cell viability. Cells were seeded in 96-well plates with 1 × 10^4^ cells per well. The cells cultured under identical growth conditions were exposed with *Giardia* at different ratios (3, 5 and 10 trophozoites/cell) for different time durations (0, 3, 6, 12 and 24 h). Following adding 10 μl of the CCK-8 solution to each well of the plate, the optical density was measured at 450 nm.

### Host-parasite interactions

Caco-2 and HT-29 cells were exposed with *Giardia* trophozoites at a ratio of 1:10 for different time durations (0, 3, 6 or 12 h). IRE1 inhibitor MKC3946 (10 nM, MCE, Beijing, China), PERK inhibitor GSK2606414 (5 μM, Abmole, Shanghai, China), ATF6 inhibitor AEBSF (200 μM, Abmole, Shanghai, China), ROS inhibitor NAC (1 mM, ApexBio, Houston, TX, USA), AKT inhibitor MK2206 2HCl (50 μM, Abmole, Shanghai, China), and JNK inhibitor SP600125 (50 μM, Abcom, Atlanta, GA, USA) were used in this study. Caco-2 and HT-29 cells were pre-treated with inhibitors for 2 h, followed by exposure to *Giardia* for 6 h.

### Cell cycle analysis

Cell cycle was analyzed with propidium iodide (PI) staining by flow cytometry (34). Briefly, Caco-2 and HT-29 cells were exposed to *Giardia* for the indicated time periods. After incubation, media was removed and cells were washed three times with cold PBS to remove *Giardia* trophozoites. After this treatment, the cultures were observed by microscopy for the presence of the trophozoites. Cells were fixed with cold 70% ethanol overnight at 4°C. Cells were then washed three times with PBS and incubated with PI and RNase for 30 minutes at 37°C. Cell cycle distributions were assayed by flow cytometry on a BD FACS Canto II. The data were analyzed with Modfit LT software version 3.1 (Verity Software House, Topsham, ME, USA).

### qPCR analysis

Total RNA was extracted using the Trizol reagent (Invitrogen, Carlsbad, CA, USA). The quality of RNA was determined by the ratios 260 nm/280 nm. Then cDNA was synthesized from 1 μg of total RNA using a HiScript II 1st Strand cDNA Synthesis Kit (Vazyme, Nanjing, China) as instructions. qPCR was performed using SYBR Green qPCR Master Mix (Bimake, Houston, TX, USA). Primer sets (**Supplementary Table 1**) used for qPCR analysis were designed and synthesized by Sangon Biotech (Shanghai, China). GAPDH was an internal control and chosen for normalizing, and the 2^−ΔΔCt^ method was used to calculate the relative expression of genes.

### Western blot analysis

Western blotting was performed as previously described (36). Equal amounts of protein per sample were separated by 12% SDS-PAGE and transferred to the PVDF membranes (Thermo Fisher Scientific, Waltham, MA, USA). Protein bands were visualized with an enhanced chemiluminescence assay kit (Syngene, Cambridge, UK), and signal intensity of protein bands was analyzed by the use of Image J software. The immunoblots were incubated with primary antibodies against cyclin A (1:1000 dilution in PBST), cyclin B (1:1000), cyclin D (1:500), cyclin E (1:750), CDK1 (1:500), CDK2 (1:1500), CDK4 (1:500), CDK6 (1:500), E2F1 (1:500), E2F2 (1:1000), E2F3 (1:500), E2F4 (1:1000), RB (1:500), p21 (1:1000), p27 (1:1000), p53 (1:500), GRP78 (1:2000), IRE1 (1:500), p-IRE1 (1:500), XBP1s (1:1000), PERK (1:1000), p-PERK (1:500), ATF4 (1:1000), CHOP (1:500), ATF6 (1:500), Skp2 (1:500), pro-CASP3/cl-CASP3 (1:500), pro-PARP/cl-PARP (1:500), AKT (1:500), p-AKT (1:500), JNK (1:500), p-JNK (1:500) and β-actin (1:8000) probed with the secondary antibody HRP conjugated goat anti-rabbit IgG (1:5000; Bioss, Beijing, China), and visualized by diaminobenzidine (DAB) (ZSGB-BIO, Beijing, China). Primary antibodies were obtained from two commercial sources (ABclonal, Wuhan, China; Bioss, Beijing, China).

### Acridine orange/ethidium bromide assay

Caco-2 and HT-29 cells were seeded at a density of 1 × 10^5^ cells/well in 24-well plates. Cells were exposed to *Giardia* for the indicated time periods. After incubation, media was removed and cells were washed three times with cold PBS. After confirmation of parasite removal by microscopy, the apoptosis of cells induced by *Giardia* was evaluated by dual staining with fluorescent dyes acridine orange (AO) and ethidium bromide (EB) (BestBio, Shanghai, China). Cells were observed under a Lionheart FX Automated Microscope (BioTek, Winooski, VT, USA) and photographed at 4× magnification. Dead or late apoptotic cells (with compromised membranes) stain orange-red, while normal cells stain green. Image J software (National Institutes of Health, Bethesda, MD, USA) was used to quantify fluorescence intensities.

### Co-immunoprecipitation assay

Protein-protein interactions were analyzed by co-immunoprecipitation (co-IP) as previously described (37). In brief, anti-E2F1 antibody and protein A/G magnetic beads (Bimake, Houston, TX, USA) were used to immunoprecipitate RB in cell lysates overnight at 4°C. Then the immunoprecipitated beats were collected and washed three times with lysis buffer. The proteins were separated by SDS-PAGE and analyzed by western blotting using anti-RB antibody.

### *In vitro* siRNA interference assay

Caco-2 and HT-29 cells were seeded at a density of 2 × 10^5^ cells/well in 12-well plates for Skp2 knockdown, cells were transfected with scramble control siRNA (si-NC) or siRNA targeting Skp2 (50 nM, Comate, Changchun, China) using lipo3000 (Invitrogen, Carlsbad, CA, USA) according to the instructions. Total RNA and protein were extracted after 48 h of transfection.

### ROS determination

Intracellular ROS was measured using the ROS assay kit (Beyotime, Shanghai, China). In brief, cells were stained with the 2’,7’-dichlorofluorescin diacetate (DCFH-DA) at 37°C for 30 min, washed with PBS for three times. The fluorescence intensity of DCF was detected by the Lionheart FX Automated Microscope mentioned earlier and photographed at 20× magnification, and also measured with a FLUOstar Omega reader (BMG Labtech GmbH, Ortenberg, Germany).

### Statistical analysis

Data distribution was assumed to be normal. Statistical analysis was completed using GraphPad Prism software v8.0 (GraphPad Software; La Jolla, CA, USA). Data were expressed as mean ± SD. The statistical significance of the differences between two groups was measured by an unpaired Student’s *t* test, and among three or more groups by one-way ANOVA. A value of *p* < 0.05 was considered statistically significant (*p < 0.05, **p < 0.01).

## Results

### *Giardia* reduced host cell viability and induced G0/G1 cell cycle arrest and apoptosis

**Figure 1:**
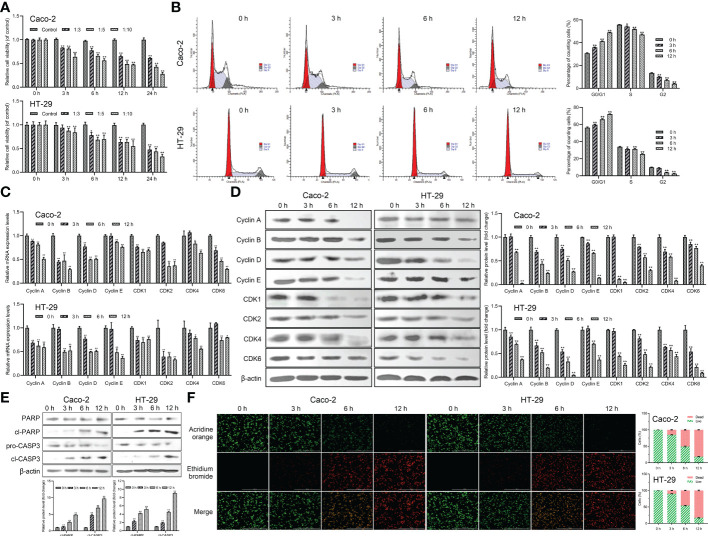
*Giardia* reduced host cell viability and induced G0/G1 cell cycle arrest and apoptosis. **(A)** Inhibition of cell viability by *Giardia* exposure in Caco-2 and HT-29 cells. Cell viability was detected *via* CCK-8 assay (n = 5 wells/group). Cells to *Giardia* ratio was 1:3, 1:5 or 1:10. **(B)** PI staining followed by flow cytometry was used to detect cell cycle distribution after the exposure of *Giardia* with 0, 3, 6, 12 h in Caco-2 and HT-29 cells. G0/G1, S and G2 indicate the different phases of the cell cycle. **(C)** The qPCR analysis (n = 3) was used to measure the mRNA levels of cyclins and CDKs with *Giardia* exposure in Caco-2 and HT-29 cells. The relative amounts of mRNA were normalized against GAPDH mRNA and expressed relative to the mRNA abundance in control. **(D)** Western blot analysis was used to measure the protein levels of cyclins and CDKs in Caco-2 and HT-29 cells treated with *Giardia*. **(E)** Western blotting was used to measure the expression levels of cleaved CASP3 and cleaved PARP in Caco-2 and HT-29 cells treated by *Giardia*. **(F)** Apoptotic effects of Giardia exposure on Caco-2 and HT-29 cells as assessed by AO/EB staining (n = 3 wells/group, scale bar = 1000 μm). The fluorescence intensity was quantified using Image J. All experiments were repeated at least three times. Data were presented as mean ± SD. *p < 0.05, **p < 0.01 versus relative control group. **(D, E)** Signal intensity of protein band was analyzed by using Image J software. The results of western blot analyses (n = 3) were normalized against the level of β-actin. **(B, D–F)** Representative pictures are shown.

The effect of *Giardia* on the cell viability of Caco-2 and HT-29 cells was evaluated by CCK-8 analysis. The results indicated that the cell viability of both of the Caco-2 and HT-29 cell lines was decreased in a dose-dependent manner following *Giardia* exposure and bottomed out at a ratio of 10 parasites/cell (thus was applied hereinafter) (**Figure 1A**). The cell cycle distribution after *Giardia* exposure for 0, 3, 6 and 12 h was detected using PI staining and flow cytometry. As shown in **Figure 1B**, the percentage of cells in the G0/G1 phase was increased, and the percentage of cells in the S phase was decreased following *Giardia* exposure. In order to determine the molecular mechanism of *Giardia*-induced cell cycle arrest, levels of cyclins and CDK proteins reported to play important roles in cell cycle progression were determined by qPCR and western blot analysis. The mRNA and protein expression levels of cyclins (cyclin A, cyclin B, cyclin D, and cyclin E) and CDK proteins (CDK1, CDK2, CDK4 and CDK6) showed a decreased trend at 6 h with *Giardia* exposure in both Caco-2 and HT-29 cells (**Figures 1C, D**). The effect of *Giardia* on the cell apoptosis was determined by detecting apoptosis markers (cleaved CASP3 and cleaved PARP). As shown in **Figure 1E**, CASP3 was cleaved, and CASP3-mediated PARP was also cleaved subsequently after *Giardia* exposure. Furthermore, AO/EB double staining test was used to investigate the effect of *Giardia* on Caco-2 and HT-29 cells. *Giardia* -treated groups displayed more apoptotic cells compared to the controls (**Figure 1F**).

### *Giardia* exposure arrested cell cycle by increasing expressions of p21 and p27 and promoting E2F1-RB complex formation

**Figure 2:**
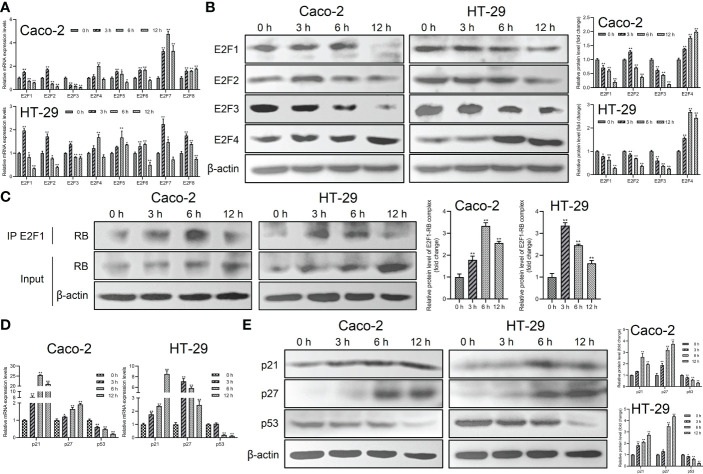
The expressions of p21 and p27 were increased and E2F1-RB complex formation was promoted by *Giardia* exposure. **(A)** The mRNA levels of E2F1-E2F8 with *Giardia* exposure in Caco-2 and HT-29 cells were measured by qPCR analysis (n = 3). **(B)** The protein levels of E2F1-E2F4 in Caco-2 and HT-29 cells treated with *Giardia* were analyzed by western blot analysis. **(C)** The E2F1-RB complex was detected by co-IP. Both IP and Input samples were analyzed by SDS-PAGE and immunoblotting. **(D)** The qPCR analysis (n = 3) was used to measure the mRNA levels of p21, p27 and p53 with *Giardia* exposure in Caco-2 and HT-29 cells. **(E)** Western blot analysis was used to measure the protein levels of p21, p27 and p53 with *Giardia* exposure in Caco-2 and HT-29 cells. All experiments were repeated at least three times. Data were presented as mean ± SD. *p < 0.05, **p < 0.01 versus relative control group. **(A, D)** The relative amounts of mRNA were normalized against GAPDH mRNA and expressed relative to the mRNA abundance in control. **(B, C, E)** Representative images are presented. The results of western blot analyses (n = 3) were normalized against the level of β-actin.

To further investigate the mechanism of *Giardia* on the cell cycle arrest, the expressions of E2Fs, p21 and p27 were examined in Caco-2 and HT-29 cells exposed to *Giardia*. For the mRNA level, E2F1, E2F2 and E2F3 was decreased at both of the 6 h and 12 h post-exposure, E2F4-E2F8 of which was increased at 6 h post-exposure (**Figure 2A**). For the protein expression level, E2F1 and E2F3 was decreased after *Giardia* exposure. The expression of E2F2 was increased at 3 h and then decreased at 6 h and 12 h under *Giardia* exposure in Caco-2 cells, but the expression of E2F2 was decreased in HT-29 cells. The expression of E2F4 was increased after *Giardia* exposure (**Figure 2B**). Although the mRNA and protein expression levels of E2Fs varied at different time points between Caco-2 and HT-29 cells, the expressions of E2Fs were indeed changed due to the exposure with *Giardia*. To assess whether *Giardia* exposure could increase the E2F1-RB interaction resulting low levels of E2F1, immunoprecipitation of cell extracts was conducted with E2F1 and RB polyclonal antibodies. The expression of RB in E2F1 immunoprecipitates was increased after *Giardia* exposure (**Figure 2C**). To determine if *Giardia*-induced G0/G1 cell cycle arrest was regulated by p21 and p27 expressions in Caco-2 and HT-29 cells, mRNA and protein levels of p21 and p27 were tested. The results showed that mRNA and protein levels of p21 and p27 were both increased with *Giardia* exposure (**Figures 2D, E**). The mRNA and protein levels of p53 were decreased with *Giardia* exposure (**Figures 2D, E**), suggesting that the p21 or p27 induction is probably p53-independent.

### The UPR and ERAD pathways were activated by *Giardia* exposure

**Figure 3:**
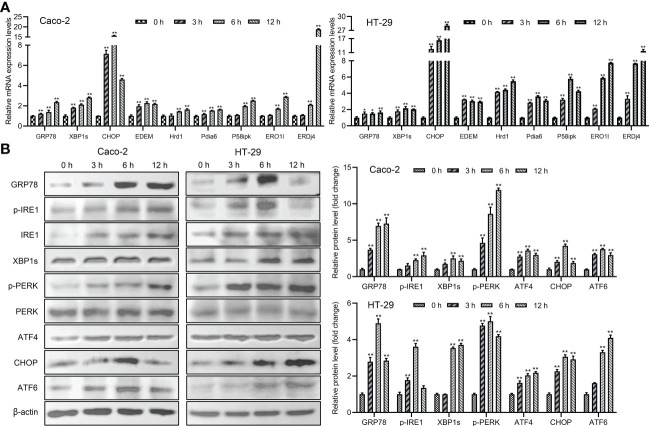
The UPR and ERAD pathways were activated by *Giardia* exposure. **(A)** The mRNA levels of ER stress-related genes and ERAD-related genes with *Giardia* exposure in Caco-2 and HT-29 cells were analyzed by qPCR analysis (n = 3). The relative amounts of mRNA were normalized against GAPDH mRNA and expressed relative to the mRNA abundance in control. **(B)** Western blot analysis (n = 3) was used to measure the expression levels of ER stress-related proteins with *Giardia* exposure in Caco-2 and HT-29 cells. Signal intensity of protein band was analyzed by using Image J software. The results of western blot analyses were normalized against the level of β-actin. All experiments were repeated at least three times. Data were presented as mean ± SD. *p < 0.05, **p < 0.01 versus relative control group.

The main proteins related to ER stress were assessed in order to determine whether ER stress could be induced and UPR and ERAD pathways were activated by *Giardia* exposure. The results showed that the mRNA levels of ER chaperone proteins and ERAD-related genes were increased with *Giardia* exposure (**Figure 3A**). As shown in **Figure 3B**, ER stress signaling pathways were significantly activated by *Giardia* exposure, as demonstrated by the increased expression levels of GRP78 and CHOP. Three ER stress signaling pathways IRE1-XBP1s, PERK-ATF4 and ATF6 were activated at 6 h post-exposure. It was demonstrated by higher-level expressions of XBP1s, ATF4 and ATF6, and activation of IRE1 and PERK phosphorylation following *Giardia* exposure (**Figure 3B**). IRE1-XBP1s is active in the adaptive phase and attenuated in the apoptotic phase (10). This might be the reason for the decrease in p-IRE1 expression in HT-29 cells at 12 h after exposure. In all, these findings suggest that *Giardia* exposure activated three UPR pathways (IRE1-XBP1s, PERK-ATF4 and ATF6) and ERAD pathway.

### The activation of UPR pathways regulated expression levels of E2Fs and formation of E2F1-RB complex to affect cell cycle progression

**Figure 4:**
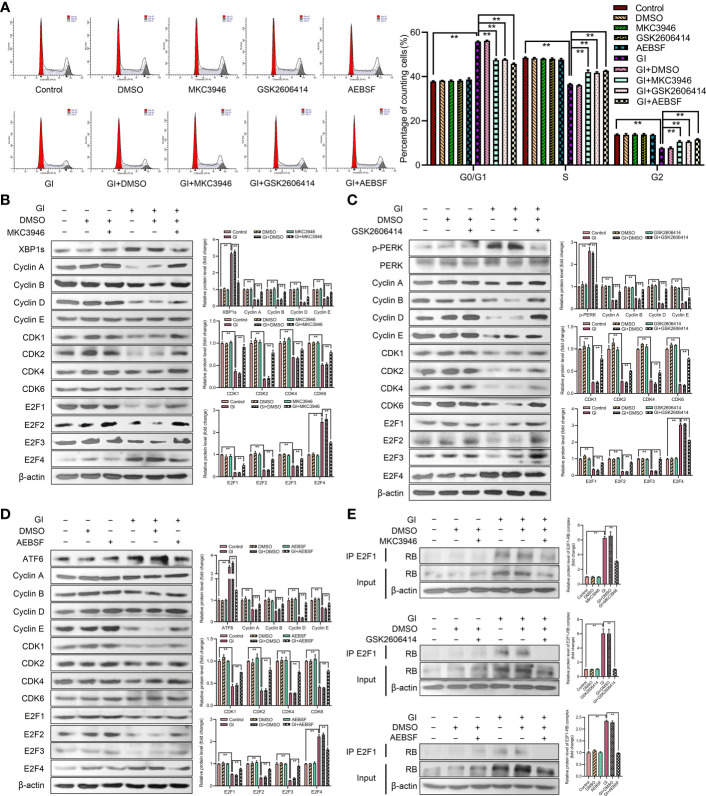
*Giardia* arrested cell cycle by activating three UPR signaling pathways. **(A)** PI staining followed by flow cytometry was used to detect cell cycle distribution in Caco-2 cells treated with or without inhibitors accompanied by *Giardia* exposure for 6 h; IRE1 inhibitor (MKC3946), PERK inhibitor (GSK2606414) and ATF6 inhibitor (AEBSF). G0/G1, S, and G2 indicate the different phases of the cell cycle. Under treatment of the combination of *Giardia* with **(B)** inhibitor MKC3946, **(C)** GSK2606414 or **(D)** AEBSF in Caco-2 cells, the expressions of cyclins, CDKs and E2Fs were detected by western blotting. **(E)** Co-IP experiments were performed to detect E2F1-RB complex formation in Caco-2 cells treated with or without inhibitors accompanied by *Giardia* exposure for 6 h; IRE1 inhibitor (MKC3946), PERK inhibitor (GSK2606414) and ATF6 inhibitor (AEBSF). Gl, *Giardia*. All experiments were repeated at least three times. Data were presented as mean ± SD. *p < 0.05, **p < 0.01 versus relative control group. **(B–E)** Signal intensity of protein band was analyzed by using Image J software. The results of western blot analyses (n = 3) were normalized against the level of β-actin. **(A–E)** Representative images are presented.

To further investigate the role of UPR signaling pathways in cell cycle progression, the effects of cell cycle and the expression levels of G0/G1 phase-associated cyclins and CDKs were determined after treatment by combination of inhibitors (IRE1 inhibitor MKC3946, PERK inhibitor GSK2606414, ATF6 inhibitor AEBSF) and *Giardia*. As compared with cells treated by *Giardia* alone, the combination of inhibitors with *Giardia* significantly decreased the percentage of G0/G1 phase (**Figure 4A**), whereas increased the expression of cyclins, CDKs and E2F1-E2F3, and downregulated the expression of E2F4 (**Figures 4B–D**), but inhibitors alone had no effects on the cell cycle. Three inhibitors of UPR pathways inhibited the formation of E2F1-RB complex after *Giardia* exposure in Caco-2 cells (**Figure 4E**).

### The activation of UPR pathways regulated the expression levels of p21 and p27

**Figure 5:**
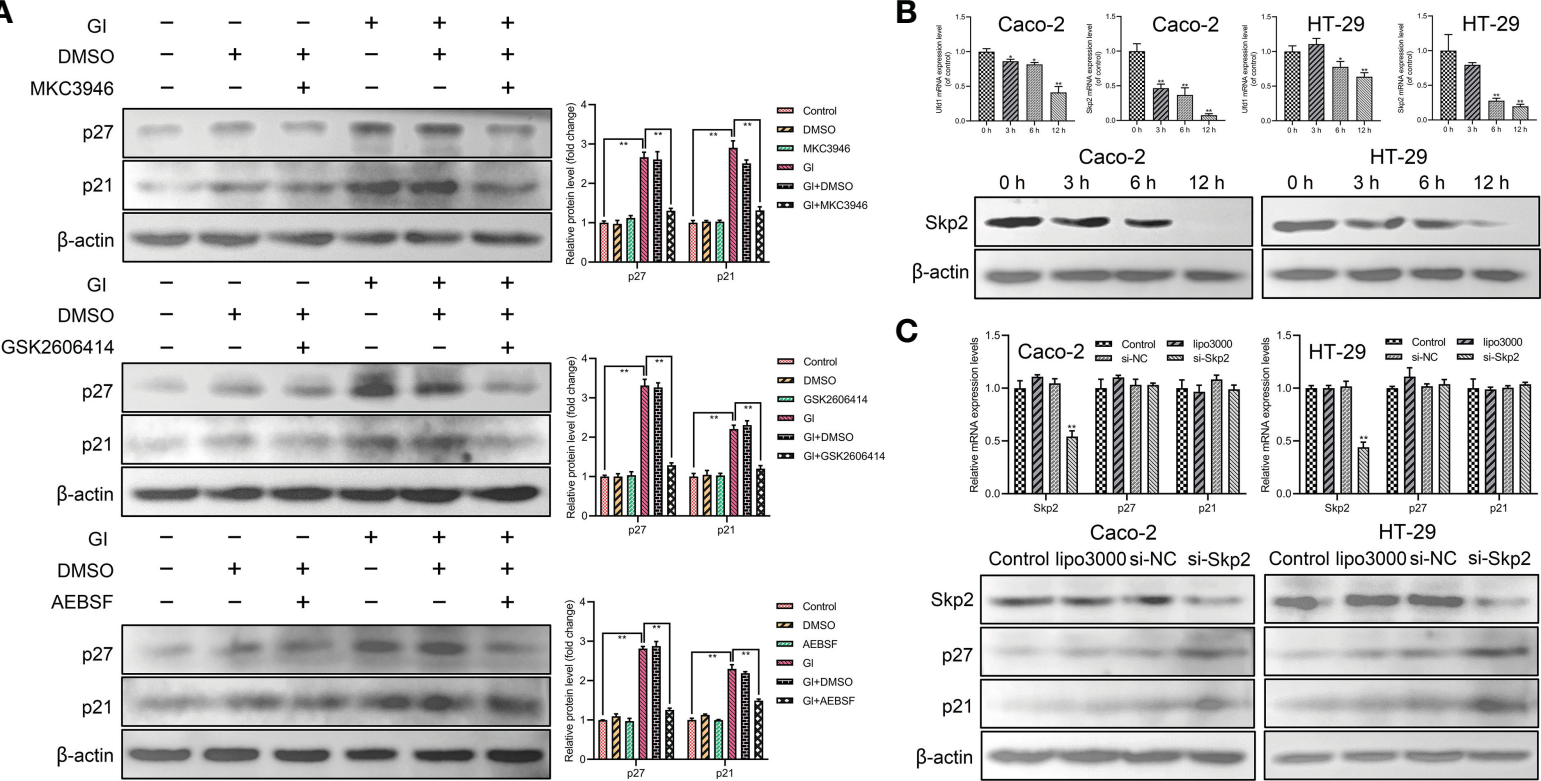
The expressions of p21 and p27 were enhanced by *Giardia* exposure *via* activating UPR pathways. **(A)** Western blot analysis (n = 3) was used to measure the protein levels of p21 and p27 in Caco-2 cells treated with or without inhibitors accompanied by *Giardia* exposure for 6 h; IRE1 inhibitor (MKC3946), PERK inhibitor (GSK2606414) and ATF6 inhibitor (AEBSF). The results of western blot analyses were normalized against the level of β-actin. Gl, *Giardia*. **(B)** The qPCR analysis was used to detect the mRNA levels of Ufd1 and Skp2 with *Giardia* exposure in Caco-2 and HT-29 cells. The protein expression of Skp2 with *Giardia* exposure in Caco-2 and HT-29 cells was detected by western blotting. **(C)** The qPCR and western blot analysis were used to measure mRNA and protein expression levels of Skp2, p21 and p27 in Caco-2 and HT-29 cells transfected with si-Skp2/si-NC for 48 h. All experiments were repeated at least three times. Data were presented as mean ± SD. *p < 0.05, **p < 0.01 versus relative control group. **(B, C)** The results of qPCR analyses (n = 3) were normalized against the level of GAPDH. **(A–C)** Representative pictures are shown.

In order to determine the relationship between UPR signaling pathways and p21 and p27 induction, MKC3946 (inhibitor of IRE1), GSK2606414 (inhibitor of PERK) and AEBSF (inhibitor of ATF6) was used to block the *Giardia*-activated UPR signaling pathways. The results indicated that the expressions of p21 and p27 were decreased when *Giardia*-induced upregulation of XBP1s, PERK and ATF6 was inhibited by MKC3946, GSK2606414 and AEBSF (**Figure 5A**), respectively. Meanwhile, the inhibitors alone had no effects on the expressions of p21 and p27. In all, p21 and p27 might be tightly linked to the activation of UPR pathways. The mRNA levels of Ufd1 and Skp2 were both decreased and the protein expression level of Skp2 was decreased under *Giardia* exposure (**Figure 5B**). To further investigate the role of Skp2 in regulating p21 and p27, siRNA was used to knockdown Skp2 and the protein expression levels of p21 and p27 were measured. Loss of Skp2 did not change mRNA levels of p21 and p27, but upregulated protein expressions of p21 and p27 (**Figure 5C**).

### ROS induced ER stress and regulated cell cycle progression

**Figure 6:**
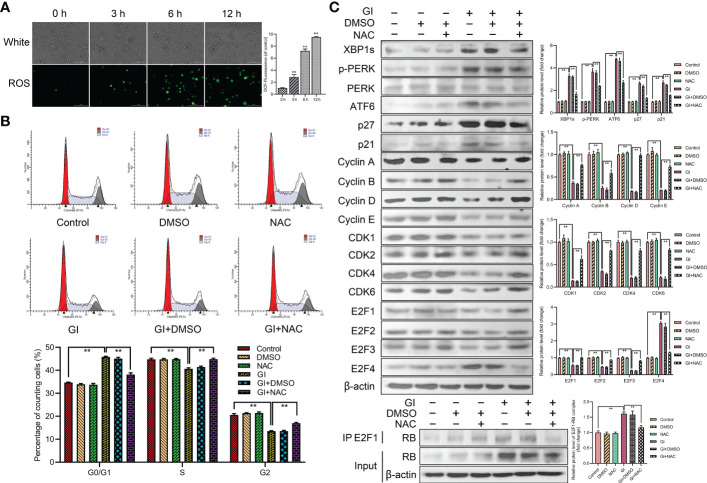
Host cell cycle arrest induced by *Giardia via* ROS‐mediated ER stress. **(A)** The ROS assay kit was used to measure ROS generation in Caco-2 cells with *Giardia* exposure for 0, 3, 6, 12 h (n = 3 wells/group, scale bar = 100 μm). Under treatment of the combination of *Giardia* with ROS inhibitor NAC in Caco-2 cells, **(B)** cell cycle distribution was detected by flow cytometry, **(C)** the expressions of UPR-related proteins, p21, p27, cyclins, CDKs and E2Fs were detected by western blotting, and formation of E2F1-RB complex was detected by co-IP. The results of western blot analyses (n = 3) were normalized against the level of β-actin. All experiments were repeated at least three times. Data were presented as mean ± SD. *p < 0.05, **p < 0.01 versus relative control group. **(A–C)** Representative pictures are shown. G0/G1, S, and G2 indicate the different phases of the cell cycle. Gl, *Giardia*.

As a significant regulator of several physiological and pathophysiological processes, ROS is essential for cell proliferation, survival and apoptosis. The study indicated that ROS level was increased during *Giardia* exposure (**Figure 6A**). Furthermore, ROS was examined for its role in *Giardia*-induced ER stress and cell cycle arrest. The percentage of G0/G1 phase was significantly decreased by the combination of NAC (ROS inhibitor) with *Giardia* (**Figure 6B**), but inhibitor alone had no effects on cell cycle. As shown in **Figure 6C**, the expressions of UPR-related proteins, p21, p27 and E2F4, were decreased by the combination of NAC with *Giardia*. The expressions of cyclins, CDKs and E2F1-E2F3 were increased and E2F1-RB complex formation was reduced by the combination of NAC with *Giardia* (**Figure 6C**).

### The activation of UPR pathways regulated apoptosis induced by *Giardia*

**Figure 7:**
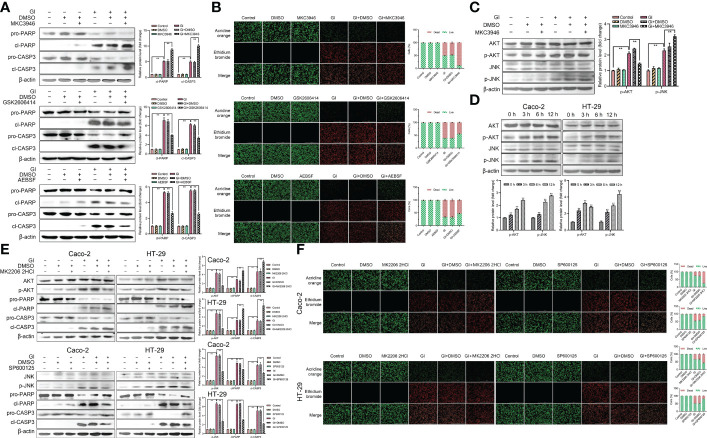
Host cell apoptosis was regulated by *Giardia* exposure *via* activating three UPR signaling pathways. **(A)** Western blot analysis was used to measure the protein levels of cleaved CASP3 and cleaved PARP in Caco-2 cells treated with or without inhibitors accompanied by *Giardia* exposure for 6 h; IRE1 inhibitor (MKC3946), PERK inhibitor (GSK2606414) and ATF6 inhibitor (AEBSF). **(B)** Apoptotic effects on Caco-2 cells during *Giardia* exposure under application of inhibitors (MKC3946, GSK2606414 or AEBSF) as assessed by AO/EB staining (n = 3 wells/group, scale bar = 1000 μm). The fluorescence intensity was quantified using Image J. Under treatment of the combination of IRE1 inhibitor MKC3946 with *Giardia* in Caco-2 cells, **(C)** western blot analysis was used to measure the phosphorylation levels of AKT and JNK. **(D)** The phosphorylation levels of AKT and JNK were detected by western blotting in Caco-2 and HT-29 cells with *Giardia* exposure for 0, 3, 6, 12 h. Under treatment of the combination of *Giardia* with AKT inhibitor MKC2206 2HCl or JNK inhibitor SP600125, **(E)** the expression levels of cleaved CASP3 and cleaved PARP in Caco-2 and HT-29 cells were detected by western blot analysis, **(F)** AO/EB staining was used to detect cell apoptosis. The fluorescence intensity was quantified using Image J. All experiments were repeated at least three times. **(A, C–E)** The results of western blot analyses were normalized against the level of β-actin. Data were presented as mean ± SD (n = 3). *p < 0.05, **p < 0.01 versus relative control group. **(A–F)** Representative pictures are shown. Gl, *Giardia*.

To further clarify the role of UPR pathways in apoptosis caused by *Giardia* exposure, MKC3946 (inhibitor of IRE1), GSK2606414 (inhibitor of PERK) and AEBSF (inhibitor of ATF6) were used to block the *Giardia*-activated UPR signaling pathways. The results showed that the expression levels of cleaved CASP3 and cleaved PARP were decreased in cells pre-treated with GSK2606414 and AEBSF, but were increased pre-treated with MKC3946 under *Giardia* exposure (**Figure 7A**). AO/EB double staining test was used to investigate the effect of UPR signaling on apoptosis. The result obtained from AO/EB double staining was showed in **Figure 7B**. The number of apoptotic cells was decreased by the use of GSK2606414 and AEBSF, and increased by the use of MKC3946 under *Giardia* exposure. To further investigate the mechanism of IRE1 signaling pathway regulating apoptosis, the phosphorylation levels of AKT and JNK were measured. AKT phosphorylation could be detected at a significantly low level in Caco-2 cells when MKC3946 was used. On the other hand, JNK phosphorylation level was increased in Caco-2 cells when MKC3946 was used (**Figure 7C**). As shown in **Figure 7D**, the phosphorylation levels of AKT and JNK were upregulated by *Giardia* exposure. Considering the role of the phosphorylation of AKT and JNK in regulating apoptosis, the AKT inhibitor MK2206 2HCl and JNK inhibitor SP600125 were applied. The AKT phosphorylation suppression by MK2206 2HCl upregulated the expression levels of cleaved CASP3 and cleaved PARP at least partially, and JNK phosphorylation suppression by SP600125 downregulated expression levels of cleaved CASP3 and cleaved PARP at least partially (**Figure 7E**). AO/EB double staining was used to investigate the role of AKT and JNK pathways in regulating apoptosis. AKT phosphorylation suppressed apoptosis and JNK phosphorylation promoted apoptosis induced by *Giardia* (**Figure 7F**). It is possible that the hypophosphorylation of AKT and hyperphosphorylation of JNK could be contributors to the increased *Giardia*-induced apoptosis observed in cells pre-treated with MKC3946.

## Discussion

**Figure 8:**
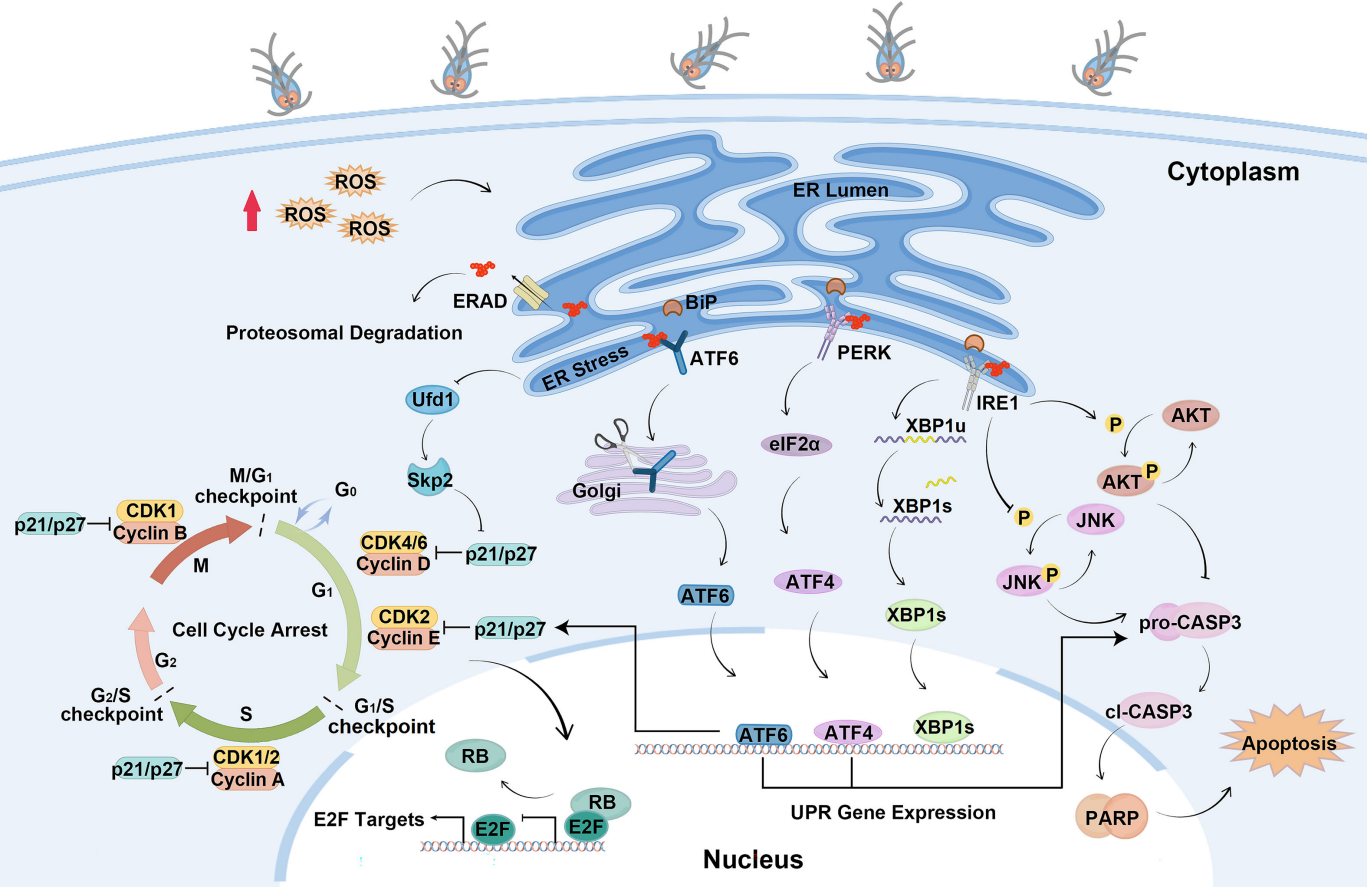
Schematic diagram illustrating the molecular mechanism of cell cycle arrest and apoptosis regulated by ER stress during *Giardia* exposure (by Figdraw, https://www.figdraw.com).

In the present study, the mechanisms of cell cycle arrest and apoptosis regulated by *Giardia*-induced UPR activation were elucidated. This study found that *Giardia* induced the UPR in a ROS-dependent fashion and the activation of UPR pathways regulated expressions of p21, p27 and formation of E2F1-RB complex to induce cell cycle arrest. PERK and ATF6 pathways promoted apoptosis, and IRE1 pathway suppressed apoptosis induced by *Giardia* through regulating phosphorylation levels of AKT and JNK (**Figure 8**).

The Caco-2 and HT-29 were intestinal cell lines derived from a colon adenocarcinoma and have been used as an enterocyte model in bioavailability and cell mechanism studies. Once differentiated, Caco-2 cells form a polarized cell monolayer with apical and basolateral membranes, a junction complex and a brush border with microvilli on the apical side typical of human enterocytes (38). HT-29 can differentiate into goblet-like cells and thus has the ability to produce mucus. Caco-2 cells and HT-29 cells were used as enterocyte models for *Giardia* infection. However, certain limitations of the models exist. Caco-2 cells are lacking for mucus layer that covers the intestinal epithelium. The HT-29 cell line displays different patterns of enterocytic differentiation in different culture media. *Giardia* colonizes the small intestine, but Caco-2 and HT-29 were derived from the human colon and the translocating properties, enzyme expressions and transmembrane electrical resistance seemed to be close to the colon rather than the small intestine.

*Giardia* induced cell cycle arrest and upregulated the expressions of p21 and p27. As p21 and p27 have been extensively characterized as negative regulators of G1-S progression (7), it is reasonable to speculate that the induction of p21 and p27 may play an important role in the cell cycle exit induced by *Giardia*. *Toxoplasma gondii* rhoptry protein ROP16 and *Eimeria tenella* ROP1 induce host cell cycle arrest through the activation of p53/p21 pathway (20, 39). The p53/p21 pathway is a classical pathway involved in cell cycle arrest, but the expression of p53 was reduced by *Giardia* infection in this study, it is likely that expressions of p21 and p27 were regulated by other pathways to induce cell cycle arrest during *Giardia* infection. Cellular homeostasis relies on E2F activity during the cell cycle (40). Certain parasites specifically target the E2Fs and perturb cell cycle progression. For instance, *Theileria* parasites affect E2F signaling to promote leukocyte proliferation (41) and *Toxoplasma gondii* targets the E2Fs to elicit a key set of host responses related to the cell cycle (42). In this study, E2F1-RB complex formation was increased and expressions of E2Fs were regulated by *Giardia* exposure. The repression of transcription of E2F1 by E2F7 and E2F8 has been demonstrated previously (16, 40). Hence, we speculate that E2F1 may be regulated by E2F7 and E2F8 during *Giardia* infection, although the precise mechanism is still unknown.

The ability to recognize cellular stress and trigger adaptive host defenses has been indispensable throughout evolution. As a cellular organelle that controls protein quality and distinguishes attacks on protein fidelity, the ER holds a unique position in the cell (43, 44). It has been previously shown that UPR activation regulates cell cycle progression (45, 46). In order to elucidate the mechanism of cell cycle arrest regulated by UPR activation induced by *Giardia*, the levels of UPR-related proteins and cell cycle-regulated proteins were measured in this study. Results showed that *Giardia* infection induced ER stress and UPR activation. The activation of UPR pathways may be closely correlated with the expressions of p21 and p27. Some studies have reported that the activation of ER stress signaling pathway IRE1-XBP1s induces cell cycle arrest by increasing expressions of p21 and p27 (7). The activation of PERK/eIF2α signaling has been shown to cause loss of cyclin D1, resulting in G1 cell cycle arrest (6, 47). However, the current study does not demonstrate a direct association of activation of ATF6 signaling pathway with cell cycle progression. It has been previously shown that crosstalk between these different UPR signaling pathways (46, 48, 49). Therefore, we speculate that the UPR will engage in complex crosstalk networks to regulate cell cycle arrest. Research has proved that the Ufd1 facilitates clearance of misfolded proteins through the ERAD pathway. Downregulation of Ufd1 leads to increased ubiquitination and destabilization of Skp2, resulting in accumulation of p27 and a concomitant delay in the G1 phase of the cell cycle (50). The mRNA levels of Ufd1 and Skp2 were decreased during *Giardia* infection, and Skp2 regulated the expressions of p21 and p27 when judged with siRNA knockdown experiments. The mRNA levels of ERAD-related genes were increased under *Giardia* exposure. Thus, we assume that cell cycle arrest induced by UPR activation might affect ERAD.

Recent studies have reported that *Giardia* can induce apoptosis through both the intrinsic and extrinsic pathways (34, 51). *Giardia* can modulate the processes of apoptosis in the intestines of the host by DNA fragmentation, CASP3 activation, PARP degradation, and regulation of the expressions of Bcl-2 and Bax (22). The parasite may have a destructive effect on the intestinal epithelium of the host through the induction of various mechanisms, depending on the species. The UPR signaling pathways induce apoptosis has been documented (52, 53). The results of this study showed that PERK and ATF6 pathways involved apoptosis activated by *Giardia*. However, the activation of IRE1-XBP1s partially suppressed apoptosis induced by *Giardia*. One of the possible causes of such difference is that stress sensors have distinct sensitivities to specific inducers of ER stress. The activation of PERK inhibits general protein translation through the phosphorylation of eIF2α under mild ER stress (54). Under prolonged stress, the activation of PERK and ATF6 pathways increases expression of CHOP to initiate the apoptotic response (49, 55). It is possible that activation of PERK and ATF6 pathways regulated expression of CHOP to regulate apoptosis induced by *Giardia*. The inhibition of AKT by inhibitor MK2206 2HCl increased *Giardia*-induced PARP and CASP3 cleavage in this study. AKT hypophosphorylation in cells resulting from inhibition of the IRE1-XBP1s pathway by MKC3946 was associated with enhanced *Giardia*-induced apoptosis. The kinase activity of IRE1 has not been reported to directly phosphorylate AKT. AKT-related signaling regulated by the IRE1 pathway requires further investigation. The inhibition of JNK by inhibitor SP600125 reduced *Giardia*-induced PARP and CASP3 cleavage, and inhibition of IRE1 signaling pathway by inhibitor MKC3946 could increase phosphorylation of JNK in this study. Although several studies have documented that IRE1 activated JNK pathway to induce apoptosis (53, 56), the enhanced *Giardia*-induced apoptosis was associated with hyperphosphorylation of JNK in cells caused by inhibition of IRE1-XBP1s pathway *via* application of MKC3946 in this study. Due to this, it is likely that other upstream kinases activated the JNK pathway during *Giardia* infection, which was modulated by the uncharacterized activity of IRE1. It is also likely that AKT, JNK and IRE1 signal crosstalk may be complicated.

In conclusion, the results of this study showed that *Giardia* activated UPR signaling pathways to regulate cell cycle arrest and apoptosis. It seems that the arrested cycle is not just a passive response that allows cells to slowly return to homeostasis, but also creates conditions that allow misfolded proteins to degrade. Activation of PERK and ATF6 pathways induced apoptosis, but activation of IRE1-XBP1s pathway suppressed apoptosis to promote cell survival. This study provides new insights for understanding the pathogenesis of giardiasis and the interaction between *Giardia* and the intestinal epithelium.

## Data availability statement

The original contributions presented in the study are included in the article/**Supplementary Material**. Further inquiries can be directed to the corresponding authors.

## Author contributions

Conceptualization: SY, WL. Funding acquisition: WL. Investigation: SY, XQ, HZ. Methodology: SY. Project administration: WL. Resources: WL, XL. Supervision: WL. Validation: WL. Writing – original draft: SY. Writing – editing: JG. Writing – review: WL. All authors contributed to the article and approved the submitted version.
