# Supplementary material for: Giardia duodenalis-induced G0/G1 intestinal epithelial cell cycle arrest and apoptosis involve activation of endoplasmic reticulum stress in vitro
Source: Front Immunol. 2023 Mar 15;14:1127552. doi: 10.3389/fimmu.2023.1127552 (PMC10050679; doi:10.3389/fimmu.2023.1127552)
Supplement: Supplementary file 1 [file Table_1.pdf]

Table S1. Primer pairs used in qPCR analysis.

Gene	Primer sequence (5'-3')	Accession number	Product size
GAPDH	F: CAGGAGGCATTGCTGATGAT<br>R: GAAGGCTGGGGCTCATTT	NM_001357943.2	138 bp
cyclin A	F: CGCTGGCGGTACTGAAGTC<br>R: GAGGAACGGTGACATGCTCAT	NM_001237.5	120 bp
cyclin B	F: AATAAGGCGAAGATCAACATGGC<br>R: TTTGTTACCAATGTCCCCAAGAG	NM_001354845.2	111 bp
cyclin D	F: CAATGACCCCGCACGATTTC<br>R: CATGGAGGGCGGATTGGAA	NM_053056.3	146 bp
cyclin E	F: TTGTGTCCTGGCTGAATGTATA<br>R: AAGGAAATTCAAGGCAGTCAAC	XM_011527440.3	150 bp
CDK1	F: CACAAAACTACAGGTCAAGTGG<br>R: GAGAAATTTCCCGAATTGCAGT	XM_005270303.4	94 bp
CDK2	F: CCAGGAGTTACTTCTATGCCTGA<br>R: TTCATCCAGGGGAGGTACAAC	NM_052827.4	90 bp
CDK4	F: ATGGCTACCTCTCGATATGAGC<br>R: CATTGGGGACTCTCACACTCT	NM_000075.4	124 bp
CDK6	F: TCTTCATTCACACCGAGTAGTGC<br>R: TGAGGTTAGAGCCATCTGGAAA	XM_047419716.1	130 bp
p21	F: GATGGAACTTCGACTTTGTCAC<br>R: GTCCACATGGTCTTCCTCTG	NM_001374511.1	194 bp
p27	F: CTAACTCTGAGGACACGCATTT<br>R: TTGAGTAGAAGAATCGTCGGTT	NM_004064.5	122 bp
p53	F: TTCCTGAAAACAACGTTCTGTC<br>R: AACCATTGTTCAATATCGTCCG	NM_001407269.1	85 bp
E2F1	F: ATAGTGTCACCACCACCATCAT<br>R: GAAAGGCTGATGAACTCCTCAG	NM_005225.3	221 bp
E2F2	F: ATGGTACGGGTGAGGAGTGGATAAG<br>R: CTTGTGAGAGGTCAGAAGTCAGAAGAC	XM_005245749.4	102 bp
E2F3	F: GTCATCAGTACCTCTCAGATGG<br>R: GCAGACCAAGAGACGTATCATA	XM_0047418266.1	131 bp
E2F4	F: GAAATCTTTGATCCCACACGAG<br>R: ACTCTCGTCCAGGTTGTAGATA	NM_001950.4	138 bp
E2F5	F: CTCACTACCAAGTTCGTGTCG<br>R: TTTTGCCTCACAGCCAAAGTAT	NM_001083588.2	92 bp
E2F6	F: GCCTCAATGCTTTTCCTCCCTCTG<br>R: TGCTCCTCCTCCTGTATTCCCTATTC	XM_047443601.1	133 bp
E2F7	F: CGTCAGGGTCAGGGTCAGAGAG<br>R: TTAGTGGCTGGCTCATCCTCCTC	XM_011537969.3	136 bp
E2F8	F: CAAACCACAGGATTTACAGCTC<br>R: CCATTAGCTTCAACGGTGTTAC	XM_047427597.1	111 bp
GRP78	F: CACGGTCTTTGACGCCAAG<br>R: CCAAATAAGCCTCAGCGGTTT	NM_005347.5	215 bp
XBP1s	F: TGGATGCCCTGGTTGCT<br>R: CACCTGCTGCGGACTCA	NM_001393999.1	87 bp
CHOP	F: GGAAACAGAGTGGTCATTCCC<br>R: CTGCTTGAGCCGTTCATTCTC	XM_047428446.1	116 bp
EDEM	F: CAAACATTCGAGTGGTAGGAGG<br>R: CGCCATGAAGTAAGTTCACTGT	NM_001355008.2	192 bp
Hrd1	F: GCTCACGCCTACTACCTCAAA<br>R: GCCAGACAAGTCTCTGTGACG	XM_047427713.1	215 bp
Pdia6	F: AGGAGGTCAGTATGGTGTTCAG<br>R: GAGGCGATCCTTCACGAGC	NM_001282707.2	151 bp
P58ipk	F: CGTTCACAAGCACTTAACGCT<br>R: GTTCTGCATCCCAAACACAAAC	NM_006260.5	97 bp
ERO1L	F: AGTAACCACTAACCTGGCAGA	NM_001382467.1	118 bp

	R: GGCTGGGGATTCTTGTTTGG		
ERDj4	F: TCTTAGGTGTGCCAAAATCGG	NM_012328.3	81 bp
	R: TGTCAGGGTGGTACTTCATGG		
Ufd1	F: GAGGGAAGATAATTATGCCAC	NM_001035247.3	189 bp
	R: CTTCCAAGAGTAAGTTCTGC		
Skp2	F: GCTATGCACAGGAAGCACCT	XM_047417536.1	295 bp
	R: CCCATGAAACACCTGGAAAG		
